# Perceptions of physical activity among elderly residents and professionals in assisted living facilities

**DOI:** 10.1186/s11556-017-0171-9

**Published:** 2017-02-11

**Authors:** Annsofie Mahrs Träff, Elisabet Cedersund, Catharina Nord

**Affiliations:** 1 Department of Social and Welfare Studies, National Institute for the Study of Ageing and Later Life, Division of Ageing and Social Change, Linköping university, 60174 Norrköping, Sweden; 2 Department of Spatial Planning, Faculty of Engineering, Blekinge Institute of Technology, 371 79 Karlskrona, Sweden

**Keywords:** Older people, Residential care, Qualitative interviews, Phenomenography, Variation of perceptions, Descriptive categories

## Abstract

**Background:**

Physical activity is often described as being important for people of all ages, but what different people mean when they talk about physical activity is unclear.

**Method:**

A phenomenographic method was used to analyze how 13 older people and 17 professionals answer the question, “If I say physical activity, what does the concept mean to you?” as part of semi-structured interviews conducted in four assisted living facilities in two different municipalities.

**Results:**

We identified a number of different perceptions of physical activity, with the older people and professionals having different responses. Elderly and professionals alike, define physical activity as a requirement for life and as an opportunity to maintain the body although they define the concepts in different ways. Elderly define the concept as a way to create meaning and the professionals have the attitude that the concept means everyday activities.

**Conclusion:**

The concept of physical activity may be defined in many different ways. This study has shown that elderly and professionals do not define physical activity in the same way. Therefore, professionals need to be aware of these differences when talking with elderly about individual needs in everyday life.

## Background

Older people represent a growing subset of the population [1, 2]. For some older people, aging involves a move to assisted living when their physical or cognitive abilities are no longer sufficient for them to live on their own. Most people who live in assisted living facilities are in the fourth age, which constitutes the last phase of life according to Laslett [3, 4]. Persons in the fourth age usually have multiple diseases and severe disabilities, and are in need of much care and attention, to which the assisted living facility caters.

Today’s society often emphasizes how important it is for people to be physically active in order to maintain and enhance the body’s strength and function. This emphasis on physical activity is often presented in terms of the maintenance of good health, and this applies to people in general as well as those in specific life circumstances and of different ages [1, 5, 6]. Older people in assisted living should be regarded as full members of society like everyone else [2] and should be able to participate in everyday life and make their own choices; even into assisted living, he or she should have the possibility of making decisions about their own lives. Therefore, it is important to understand how the need for physical activity is understood and addressed among elderly in assisted living facilities.

Several researchers have attempted to define the concept of physical activity. The most common definition of physical activity is any effort produced by skeletal muscles that requires energy consumption over a basal metabolic level [7]. This definition embraces both theoretical and practical dimensions *and is used in both research and policy-making* [1, 6–8]. However, there are differences between physical activities such as exercise and physical activity in everyday activities [9, 10]. *Exercise, is* defined [7] as physical activity that is planned, structured, and carried out systematically and regularly to improve or maintain *physical fitness*. In this study, we do not focus on these general definitions, but rather on people’s own definitions of physical activity and what it means to them. Most studies have explored not only older people’s perceptions of physical activity, but also their experiences, meaning-making, motivations, and hindrance barriers to physical activity [9–12]. In a few studies, the participants were presented with examples of physical activities before the interviews started [13, 14]. Physical activity has been reported to be perceived by older persons strictly as exercise and less as everyday activities [9, 10].

Physical activity is often held up as a part of everyday life and is embedded in social life [12–15]. Several studies have highlighted the social aspects, and physical activity has been suggested to improve participants’ social lives by providing opportunities to socialize with others [11, 14, 16, 17]. Social interactions during physical activities were even indicated to be more important than the activities themselves. Some studies have presented physical activity as enmeshed with older people’s everyday lives, and physical activity was indicated by participants to be an integral aspect of their personal identities [17].

Older adults continue patterns of physical activity throughout their lifetime, but with adaptations due to age-related limitations [16]. Some participants have claimed that physical activity provides improved quality of life [14]. Some factors have been ascribed the potential of having both positive and negative impacts on the level of physical activity [15]. Physical activity is seen as part of a generally healthy lifestyle [18] and is often linked to the prevention of disease and to sustained independence [12, 13, 17]. However, most studies that interpret physical activity for elderly have been performed among people who do not live in assisted living facilities. To the best of our knowledge, no study has yet examined the perceptions of physical activity among elderly living in assisted living facilities.

Professionals play a very important role in supporting older individuals to pursue an active lifestyle or to start a new habit of regular physical activity, but knowledge about professionals’ perceptions is scarce [19]. Thus, when the elderly are mostly dependent on the staff at an assisted living facility, the professionals’ perception is important. *I*t is very probable that professionals’ work to engage older people in physical activity in assisted living facilities would be facilitated by having shared perceptions of physical activity with the residents [20].

The overall aim of this study was to explore how physical activity is perceived by elderly persons and professionals in assisted living facilities. The variation of perceptions among and within these two groups were of particular interest. Therefore, we examined how both older people and professionals in assisted living facilities define the concept of physical activity, as well as which activities are being referred to when talking about physical activity.

### Conceptual levels

The decontextualized definition of physical activity takes a first-order perspective [21] that is based on statements that people make. In the second-order perspective, the statements are recontextualized and grouped into categories describing the perceptions, beliefs, and experiences that people have with regards to physical activity [21–23]. One way to perform a phenomenographic study is to analyze interviews and collect various kinds of statements. The purpose is to identify perceptions and examine how these perceptions relate to each other within common descriptive categories. The relationships between categories can be described inside the phenomenography outcome space to describe the different ways in which a phenomenon is experienced. The process focuses on identifying perceptions and examining how these perceptions relate to one another. Phenomenographers have described how we often take things for granted, which means that we do not reflect on what things mean. In phenomenographical analyses, individuals’ perceptions are highlighted and categorized based on what unites them. It is the researcher’s task to construct the core meanings into which individual perceptions are gathered based on a common theme [22].

Knowledge can be described as the relationships between many different perceptions of the world. Our knowledge of the world is fragmented because our knowledge of anything requires it be discerned and understood as “something” when it differs from a background and is taken out of context. Phenomenographic research focuses on these ideas of “something” that we take for granted. In our everyday lives, we are not aware that our knowledge is fragmented, but we see the world as a logical and coherent whole [21]. The phenomenographic method has been criticized, especially during the 1990s, which led to the development of the theoretical foundations of the method [24]. Marton, who was one of the creators of the phenomenographic method, has agreed that there are problems with the phenomenographic approach but that it does what it claims to do, namely to find the differences in how the phenomenon is perceived [22].

## Methods

### Design

We used a phenomenographic approach to explore variations in older people’s and professionals’ perceptions of the concept of physical activity. Phenomenography is a qualitative research method developed in educational research to gain knowledge about different perceptions of a concept within a group of people. The strength of the method is its ability to define a group’s experience of a phenomenon and expose variations in such perceptions [22].

Semi-structured interviews were conducted with older people and professionals at four assisted living facilities in two different municipalities in Sweden. The individual interviews obtained each informant’s idea of the concept of physical activity by asking, “If I say physical activity, what does the concept mean to you?” The analysis presented here covers the answers to this question, and the text contains quotations from the interviews to support the analysis.

### Participants

Thirteen elderly residents of assisted living facilities were interviewed (8 women, 5 men; mean age 87 years, range 70–95 years. Even the person who had no definition of the term was included). The criteria for inclusion was that they had lived in an assisted living facility for more than 6 months and they were capable of completing the interview. Thus, the interviewees had different levels of physical function and cognitive abilities. One of the interviewees said that the meaning of the term “physical activity” was unknown to her and was not included in the analysis.

Seventeen professionals were interviewed, including five care workers, four managers of assisted living facilities, four occupational therapists, and four physiotherapists. All of the interviewed professionals were women, and they had all worked in elderly care at least 2 years (range 2–28 years) and their current unit at least 1 month (range 1 month-10 years). The number of interviews with the professional staff were not sufficient to make a comparison between professional groups; therefore, we considered all of the professionals together as a single group.

### Ethics and consent

The Regional Ethical Review Board at Linköping, Sweden, approved the study (Dnr: 2012/316-31). Each residential facility was visited by the first author (AMT) before the start of the project. Older residents and professionals were invited to an information meeting about the purpose of the study were informed that data would be collected through interviews. In a information letter as well as verbally, participants were informed that participation in this study was voluntary and that they could withdraw at any time without consequence. Written information was also sent by mail to family members or trustees of the older residents. Oral informed consent was given by residents with impaired decision-making skills would find it demanding to sign a document. Despite this agreement, if the informant expressed verbal or nonverbal discomfort during the interviews, they would immediately be canceled.

### Procedure

A total of 30 interviews were carried out during 2013–2014 in four assisted living facilities chosen based on their geographic locations (situated in both large and small municipalities and the inner city, suburban and rural areas). The interviews lasted from 30 to 90 min and discussed physical activity in addition to other issues that will be the subjects of other articles. Analysis of the interviews was conducted primarily by the first author with frequent discussions with the other two article authors.

The interviews were recorded and all audible words transcribed verbatim by the first author (AMT). All hums and longer pauses were removed to make the text more readable*.*

### Analysis

The interviews were analyzed primarily by the first author, with frequent discussions with the other two authors, in accordance with Dahlgren and Fallsberg’s [25] model. An example of the procedure is given in Table 1. The analysis started with repeated reading of the part of the interview where the respondents gave their statements about the concept of physical activity. The next step was to condense the material so that the essential elements of every interview were separated out*.* The statements were compared and related to each other so that similarities and differences could be distinguished, and the material was grouped so that similar answers were together. This step was the “articulation” step in which the essential meaning of each descriptive category was grouped such that similar dialogue sequences were captured. This meant that some parts of a statement were moved to another group*.* In the next step, “labeling” was performed and the different groups named with the core meaning of the grouped material*. In the final step,* the descriptive categories were described based on their individual significance and what each category contained and did not contain.

**Table 1:** Example how the analysis was performed

The interviewer’s question			If I say physical activity, what does the concept mean to you?	
The response (1)			Well, I think it means to move, and I think it is things like gymnastics and going around doing other things. Well, little movements in different directions and so on.	
Condensed material (2)			Moving around	
			Gymnastics	
			Going around	
			Movements in different directions	
Articulation (3) and Labeling (4)	Moving	Gymnastics	Going around	Movements in different directions
Sub-groups	Moving	Exercise	Walking	Getting fit
Description category (5)	Physical activity as a requirement for life	Physical activity as an opportunity to maintain or develop the body		

Phenomenography does not aim to describe perceptions in relation to any particular individual. It is the perception itself that is important, and each individual’s perceptions could end up in different descriptive categories. The organization of meaning-units involves a decontextualization of parts of the statements, and these parts are joined together to form a new meaning-unit through recontextualization. All statements were jointly analyzed (those made by residents and those made by the professionals), but it was continuously noted if the statements were made by professionals or by older people.

## Results

The interview material contained 71 different statements in which the interviewee responded to the question “If I say physical activity, what does this mean to you?” A total of 14 statements were given by older residents and 58 statements were given by the professionals, as several of them provided more than one statement. The material was sorted into four categories (A–D; Table 2) and given the following headings: *Physical activity as a requirement for life (A), Physical activity as an opportunity to maintain or develop the body (B), Physical activity as an attitude of the professionals (C),* and *Physical activity to create meaning (D).* Categories A-C are represented in the following quotation from an interview with one of the professionals:

**Table 2:** Summary of the results with the descriptive categories and sub-groups

	Physical activity as a requirement for life (A)			Physical activity as an opportunity to maintain or develop the body (B)				Physical activity as an attitude of the professionals (C)							Physical activity to create meaning (D)
	Everything you do	Moving	Individual activities	Group activities	Get fit	Get exercise	Walking	Massage	Daily life	Everyday rehabilitation	Getting dressed	Household chores	Personal ADL	Movement	Having something to do/Pastime
Older people	*X*	*X*	X	X	X	X	X	X	X						X
Professionals	*X*	*X*	X	X	X	X	X	X	X	X	X	X	X	X

“It depends on the context. For me, physical activity is when I am out *walking* (B) and *moving around* (A). I have dogs and I´ll be out *walking* (B), for example, or I will *go to the gym* (B) or something like that. But if you’re talking in terms of work at the assisted living facility, then I think physical activity is just about *everything you do* (A) from when you get up in the morning. It’s physical activity just to *get dressed, button up your clothes, and make sure your teeth are brushed* (C) and everything, and if you do it all yourself then you have physical activity right there. *Wheeling your wheelchair out to the dining room* (C), for example, is also a physical activity. And then there’s the *seated dancing* (B) and stuff like that, but these are more made-up activities. But it’s a big effort just to get up in the morning.”

Category D is from an interview with one of the older residents:

“What I have been interested in but never really got enough time for is textiles and *weaving* (D) and things like that…”

### Description categories

#### Physical activity as a requirement for life

This descriptive category includes all of the statements that describe how physical activity is to use the body and bodily functions in a general way. The statements contained in this group are so general that they could not be incorporated into any of the other groups. The subgroups in this descriptive category include *Everything you do,* which includes general descriptions of how to use your body, and *Moving around,* which includes general descriptions of the moving body but not for any particular purpose.

This descriptive category contained many statements made by the professionals, often describing what they believed physical activity meant for older residents or making general statements about physical activity. The older residents gave statements such as *“to do things”* and *“to move”,* both of which were also stated by the professionals. One man living at one of the assisted living facilities who sits in his wheelchair most of the day and powers it on his own with great effort said the following when asked about what the concept of physical activity means to him:

“It’s moving physically and feeling good.”

Another resident who lives at the same assisted living facility and who was relatively physically vital and could move indoors entirely without any support and usually used a walker when he went outdoors. He replied:

“Yes, physical activity is just being, so that means everything I do here.”

A professional in one of the assisted living facilities gave the following response:

“All movements you’re able to do I think is physical activity. It’s not just standing and walking, I think. Physical activity can be that I can make my head move, and I can use it if it is the only part of my whole body I can move. I’m thinking of the whole person. I mean, you’re not thinking that now we’re doing physical activity and now we’re doing something else – it comes into everything. What am I able to do and what part of my body am I able to use and do I WANT to use it?”

A resident replied:

“That I am able to move, even if it’s slow going.”

#### Physical activity as an opportunity to maintain or develop the body

This descriptive category includes all of the statements in which the participants described any sort of exercise training that is intended to maintain or enhance bodily function. The subgroups in this category include *Individual activities*, which are all of the statements that describe, for example, working out at the gym and running, *Group activities*, which are the activities that people can do together in groups, such as gymnastics and seated dancing, *Get exercise/Get fit,* which are all of the statements that describe exercise in any form and in general terms, and *Walking*, which includes various statements about walking in the sense of exercising and maintaining or increasing the body’s functionality.

Some participants directly identified what they considered physical activity to be, such as “group activities” or “a brisk walk”*.* Others described the effects of physical activity, such as a “heart-rate increasing activity”. Both professionals and older residents made certain statements that were similar, including physical activity as “training”, “exercises”, “water exercises”, “moving a bit more”, or “walking”. The older residents made significantly more statements in this descriptive category than in the other categories, and there were some statements in which both older residents and professionals shared the same view. However, for many of the statements it was not always clear if they were of a general nature or if they were specifically related to the participants themselves.

One professional answered in this way when asked what physical activity meant for her:

“Well, for myself maybe I’m thinking a bit more about training and the gym and maybe moving.”

Only the older residents described the training as lighter exercises and outdoor walking. The following exchange occurred when one resident was asked about what physical activity meant for her:

Resident: “…it’s lighter exercise.”

Interviewer: “mmm”

Resident: “When you’re this old.”

Interviewer: “And what do you think about that?”

Resident: “I think it’s good.”

The last subgroup in this description category is *Massage,* where professionals described massage as a form of physical activity. One professional described it as follows:

“I think it’s all activity involving a physical movement. I mean, like, when you’re using your body in some way. When you’re walking and moving around, and when you massage someone’s hands to get the circulation going in one’s fingers.”

#### Physical activity as an attitude of the professionals

This descriptive category includes all activities that most people do in everyday life, such as taking care of hygiene and getting dressed. There were also various descriptions of movements and walking, such as going to the dining room or to the bathroom. The subgroups in this descriptive category are *Daily life*, which includes statements that describe everyday activities such as “fetching the newspaper in the morning”, *Everyday rehabilitation,* which considers everyday activities that allow you to maintain or improve your abilities, *Household*, which includes chores such as “hanging up the laundry” or “whisking cake batter”, *Personal ADL*, which includes statements such as “eating”, “buttoning your clothes”, and “brushing your teeth”, and *Movement*, which includes such statements as “just moving your bum a bit”, “walking from the bed to the bathroom”, and “walking to and from activities”*.*

This descriptive category did not include any statements from the older residents; they were solely those of the professionals. They often made statements about what they considered physical activity for older residents, but some of the statements were also given in general terms.

One professional described physical activity as follows:

“Physical activity for me as a professional and for the older people is different. For the older people, it is activity in everyday life, everyday activities. It’s not the kind of physical training as when I go to the gym and work out. For the older people, it’s getting dressed, practicing your hygiene, and walking to and from the dining room. That is physical activity to me.”

Another professional described physical activity as follows:

“If I look at the assisted living in my professional role, of course, I can also see that participating in baking, whisking a cake batter, can be a physical activity for someone who is very inactive.”

Care staff from the same assisted living facility described physical activity as follows:

“It’s that you use the functions you are able to use. By that, I mean that you might be a hemiplegic, but you can still use the healthy side of your body. Your physical function is reduced, but you can still use the physicality you have and do the best you can with what function you have left. It doesn’t have to be walking, it could be rolling your wheelchair or using a pen – all based on your individual ability.”

#### Physical activity to create meaning

This descriptive category includes statements describing occupation/hobby activities and *Activities for having something to do/Having a pastime* as physical activity. These statements were only made by older Persons in the fourth age usually have multiple diseases and severe disabilities and are in need of much care and attention, which the assisted living Most people who live in assisted living facilities are in the fourth age, which express sentiments such as having “something to do” and described activities such as “weaving” and “writing letters”. This category does not include statements about training, even if they could be regarded as leisure-time activities. One of the older residents expressed the following regarding what physical activity is for her:

Resident: “Well, what should I pick, there are a lot to choose from.”

Interviewer: “You can give several examples if you like.”

Resident: “What I've been interested in and never really had enough time for.”

### Outcome space

We identified a number of different perceptions of the concept of physical activity. The older residents’ statements were mostly in the descriptive category *Physical activity as an opportunity to maintain or develop the body*, and only older residents’ statements were included in the descriptive category *Physical activity to create meaning*. Some of the statements made by older residents were categorized under *Physical activity as a requirement for life*.

Professionals’ statements were mostly in the categories *Physical activity as an opportunity to maintain or develop the body* and *Physical activity as an attitude of the professionals*, and only professionals gave statements in the latter category. The professionals sometimes identified what physical activity meant to them and what physical activity meant to the older residents. The older residents made no such claims in their statements; they identified physical activity in relation to the older population and sometimes made general statements. Thus, there are a variety of ways to define the concept of physical activity. Table 2 summarizes who made the statements in each descriptive category and subcategory.

**Fig. 1:**
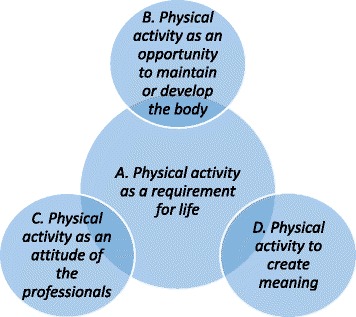
The outcome space for older people’s and professionals’ perceptions of the concept of physical activity

The descriptive categories were organized as shown in Fig. 1 to describe how the categories interact with each other. We have chosen to make descriptive category A the base category. *Physical activity as a requirement for life* is a prerequisite for the other categories because the ability to move in general terms is the basis of all other forms of physical activity [26, 27].

## Discussion

This phenomenographical study showed that there are both similarities and differences in how older and professionals perceive what physical activity means to them. A strength of this study is that we obtained statements from both older residents and staff in all professions from all of the units, making it possible to identify the differences between older people´s and professionals’ definitions at the unit level.

All of the definitions of physical activity that were given by older residents and professionals follow Caspersen et al’s [7] definition of physical activity, as they all involve skeletal muscle activity within the body. However, the distribution of statements from the two groups of participants differed substantially in certain descriptive categories. The older residents did not describe physical activity as everyday activities, but this category was the main category for the professionals’ statements, at least when they described physical activity for older persons. The professionals had an attitude that seemed to be linked to their professions; they had the task of inviting and encouraging older residents under their care to be physically active by participating in everyday activities.

Walking was expressed by many participants (both older residents and professionals) as an example of physical activity. This is in line with some previous studies in which walking was the most-mentioned physical activity [18, 28]. The results also showed that it is not obvious that everyone has a definition of physical activity.

### Older residents’ perceptions

Everyday activities were seen as physical activity in previous studies of community-living older people [13, 17], but this was not the case in our study. In two studies the participants did not mention any specific exercise, but perceived physical activity mainly as exercise [9, 10]. The results from Welmer et al’s [13] study are particularly enlightening because the participants were of a similar age range as in our study. The study defined physical activity as something you get for free when doing other things, and as such physical activity is embedded in the everyday activities of older persons. This differed from the results of the present study in which older residents defined physical activity as exercise, hobby, and pastime.

If this difference is dependent on whether the participants live in the community or a residential care facility, it suggests a number of bothersome issues related to everyday life in such care facilities. The older persons’ in the present study may have responded to the question in a way that was disconnected from their everyday life to some extent, which is different from how the care professionals responded to the question. The different results also suggest that older people living in assisted living facilities have a much more impoverished everyday life or have lost control over their life.

*When professionals pursue restorative care, do they miss out on an opportunity to engage with* older *people who want to partake in physical exercise? Older people tend to continue their habits of pursuing physical activities as when they were younger, though at an adapted level of intensity* [16]*.* The elderly care professionals must help with adaptation in terms of motivating and adapting exercise and training opportunities for the residents [29, 30].

The older residents in this study had some new ideas about what could be considered physical activity, and it is important that professionals be attentive to each individual’s own views and support the creation of physical activities in which each individual wants to participate.

In addition, the older residents in our study responded to the question in terms of what they miss or lack in terms of physical activity or what activities they desired. These results differ from the study by Welmer et al. [13], who reported how older people who lived in ordinary housing described many everyday activities in relation to physical activity and how physical activity was something they got for free. Therefore, it appears that the residential care professionals and elderly who live in their own home describe everyday activities as physical activities, but older people who live in assisted living facilities describe activities that cannot be carried out within the specific accommodation’s framework or cannot be engaged in due to physical limitations. The activities described as examples of physical activity can be performed by at least some of the older people living in assisted living facilities. Activities such as exercising at a gym and water gymnastics cannot be performed in every assisted living facility because not all of them have appropriate facilities. However, it would be possible for older people who have the opportunity to go to places where these facilities are offered to carry out such activities.

### Professionals’ perceptions

In this study the professionals were the only ones to describe physical activity as taking part in everyday activities. Professionals defined physical activity more as taking care of one’s personal hygiene or walking to the dining room to eat, which seemed to mirror a work practice similar to what Resnick et al. [19] called restorative care or function-focused care. Many of the interviewed professionals described everyday activities as the most important physical activity for older people. Larsson et al. [31] pointed out that “doings performed to manage personal and household chores need to be recognized as important doings, especially at advanced ages.”

Supporting everyday activities can be seen as part of the role of care staff and part of the culture in residential care organizations. Thus, these routines and the culture that surrounds them serve to socialize and integrate members into everyday activities. All of the rules and routines tend to be equal for all residents.

Professionals described everyday activities as a central part of physical activity for older people. If acts like performing morning bathroom chores or walking to the lunch room can be considered physical activities, then this can be a way for care staff to save time while still providing mandated physical activities for the residents. Harnett [32] described how it is usually not the lack of time that creates a lack of flexibility, but the routine culture that controls what happens within the organization of elderly care.

What are the consequences if the older person has different expectations for physical activity than the professionals? The staff members’ comments may indicate that they devalue the abilities of the older residents. This difference in perceptions between professionals and older people may be due to age discrimination or ageism [33, 34]. Ageism is prejudice or stereotypes based on a person's age and can lead to discrimination. This view may also include that you as old may be regarded as, you are weak, sick, and do not want and cannot be physically active [35]. This view could be a result of professionals not believing that older residents can or want to be physically active in ways other than through everyday activities. We suggest that research explore whether there is a discourse stipulating that older people have their physical needs fulfilled by performing various types of everyday activities. If there is such a discourse, it may affect the professionals’ daily work such that they simply do not offer opportunities for older people who want to participate in regular training. The professionals in the current study have provided rather similar definitions of physical activity. This definition is quite different from that provided by the older residents. The difference may be explained by the different roles of the two groups. The differences in expectations can in engender result in misunderstanding, frustration, disappointment, and even conflicts. As such, expectations should be taken into account in daily life.

In this study, the number of interviews was not enough to make comparisons between the different professional categories. A future study with a larger number of respondents would allow such comparisons.

### Limitations

One limitation of this study is that the education level of the staff was not surveyed beyond determining the various professional groups, who were assumed to have adequate education and training for their respective profession. Also, the level of education of the elderly participants was not identified.

Another limitation of this study is related to the fact that the interviewees only answered what the concept of physical activity means to them. The study does not show what actually happens in the assisted living facility, which activities are offered or what physical activity the individuals engage in. Answering these questions require an observational study. Another limitation may be potential bias in how the authors interpreted the answers in the analysis, but three people discussed the findings, which minimized the presence of any bias.

## Conclusion

This study showed that there are similarities and differences in how physical activity is defined and variations in the way the concept is understood. These differences and variations are apparent between older people and professionals. Elderly define the concept, among other things as hobby and pastime something that the staff did not mention. The staff however, defines the concept as everyday activities which elderly did not talk about.

Both elderly and staff define the concept as a requirement for life and as an opportunity to maintain or develop the abilities of the body. In the category Physical activity as an opportunity to maintain or develop the body, are some differences. Elderly describe physical activity in general terms, often without reference to their own training. The staff however describe physical activity as training in connection to themselves but as everyday activities for the elderly. It is important for professionals in assisted living to be aware of the different definitions. Such awareness can make professionals more confident about what older people want and what areas their individual needs and choices should apply in everyday life.
